# The *S*. *aureus* 4-oxalocrotonate tautomerase SAR1376 enhances immune responses when fused to several antigens

**DOI:** 10.1038/s41598-017-01421-z

**Published:** 2017-05-11

**Authors:** Pauline M. van Diemen, Darren B. Leneghan, Iona J. Brian, Kazutoyo Miura, Carole A. Long, Anita Milicic, Sumi Biswas, Christine S. Rollier, David H. Wyllie

**Affiliations:** 1 Jenner Institute, University of Oxford, CCMP, OX3 7BN UK; 2 Jenner Institute, University of Oxford, ORCRB, OX3 7DQ UK; 3 Laboratory of Malaria and Vector Research, National Institute of Allergy and Infectious Disease, National Institutes of Health, Rockville, Maryland USA; 4 Oxford Vaccine Group, Department of Paediatrics, University of Oxford, and the NIHR Biomedical Research Centre, CCVTM, Churchill Lane, OX37LE Oxford UK

## Abstract

A persistent goal of vaccine development is the enhancement of the immunogenicity of antigens while maintaining safety. One strategy involves alteration of the presentation of the antigen by combining antigens with a multimeric scaffold. Multi-antigen vaccines are under development, and there are presently far more candidate antigens than antigen scaffolding strategies. This is potentially problematic, since prior immunity to a scaffold may inhibit immune responses to the antigen-scaffold combination. In this study, a series of domains from *S*. *aureus* which have been shown to crystallise into multimeric structures have been examined for their scaffolding potential. Of these domains, SAR1376, a 62 amino acid member of the 4-oxalocrotonate tautomerase (4-OT) family, was pro-immunogenic in mice when fused to a range of pathogen antigens from both *S*. *aureus* and *P*. *falciparum*, and delivered by either DNA vaccination, viral vector vaccines or as protein-in-adjuvant formulations. The adjuvant effect did not depend on enzymatic activity, but was abrogated by mutations disrupting the hexameric structure of the protein. We therefore propose that SAR1376, and perhaps other members of the 4-OT protein family, represent very small domains which can be fused to a wide range of antigens, enhancing immune responses against them.

## Introduction

Modern vaccinology approaches use highly purified protein antigens which often have limited innate stimulatory activity and so may be poorly immunogenic. To address this, multiple strategies have been developed, including incorporating protein antigens into oil emulsions, formulating with aluminium salts, and addition of, or fusion to, Toll-like receptor agonists^1^. Alternatively, by using DNA or recombinant viral vectors, innate immune stimulation can be achieved by the vector while exploiting the host’s translational machinery to provide *in vivo* expression of the antigen^2^.

A separate strategy, which has a good safety profile, involves alteration of the size and multimerisation of the antigen either by attachment of the protein to a self-assembling bacteriophage or by fusion to part of a viral capsid^3–5^ or other similar protein^6^. Such virus-like particles (VLPs), which are typically 20–200 nm in diameter, include commercially deployed human papilloma virus and hepatitis B vaccines^4^ as well as numerous products at earlier stages in development^5, 7^. The varied immunological mechanism(s) behind VLP induced enhanced immunogenicity include ready access to lymphatics, rapid dendritic cell uptake and activation and arrayed-antigen mediated B-cell receptor cross-linking^4^. In some cases, particle entry into cells mediated by specific host receptors has been demonstrated^8, 9^. VLP immunogenicity is likely a result of a combination of these mechanisms.

A limited number of non-viral antigen multimerisation domains have been described. One such domain, IMX313, is derived from the multimerisation domain of a vertebrate complement C4 binding protein (C4bp)^2^, and extensively re-engineered to minimise cross-reactivity with human C4bp. Marked improvements in immunogenicity to some antigens have been observed with this strategy^10, 11^. Other related technologies include fusion to ferritin or encapsulin molecules^12^, or fusion with the highly multimerising protein lumazine synthetase^13^.

Vaccine development is continuing for a range of bacterial pathogens, including *S*. *aureus*, pathogenic *Neisseria* species^14^, *M*. *tuberculosis* ^15^, *E*. *coli* ^14^, and against *Apicomplexa* (e.g. *P*. *falciparum* ^10^). Multi-antigen vaccines are under development, and there are presently far more candidate antigens than antigen scaffolding strategies. This is potentially problematic, since prior immunity to a scaffold may inhibit immune responses to the antigen-scaffold combination, as was observed with circumsporozoite protein-hepatitis B surface antigen fusions in human adults^16^. It is at present unclear how many molecules exist biologically which are capable of enhancing immunogenicity when fused to other antigens, what the required biophysical properties are, and whether multimerisation is necessary for the adjuvanting effect. Nevertheless, if such proteins exist, pro-immunogenic domains unrelated both to mammalian proteins and to existing viral-like particle components, including Hepatitis B surface antigen, might have utility in a range of vaccines which are currently being developed.

In this study, we have examined a series of domains from *S*. *aureus* proteins which have been shown to crystallise into multimeric structures. We show that SAR1376, a member of the 4-oxalocrotonate tautomerase (4-OT) family, is pro-immunogenic in mice when fused to a range of pathogen antigens from *S*. *aureus* and from *P*. *falciparum*, whether delivered by DNA vaccination, viral vectored vaccines or as protein-in-adjuvant formulations. We demonstrate by mutagenesis that the adjuvant effect does not depend on enzymatic activity, but is abrogated by mutations unfolding the hexameric structure of the protein. We therefore propose that 4-OT proteins represent a very small pro-immunogenic domain which can be fused to a range of antigens, enhancing immune responses against them.

## Results

### Selection of scaffolding tags

**Figure 1:**
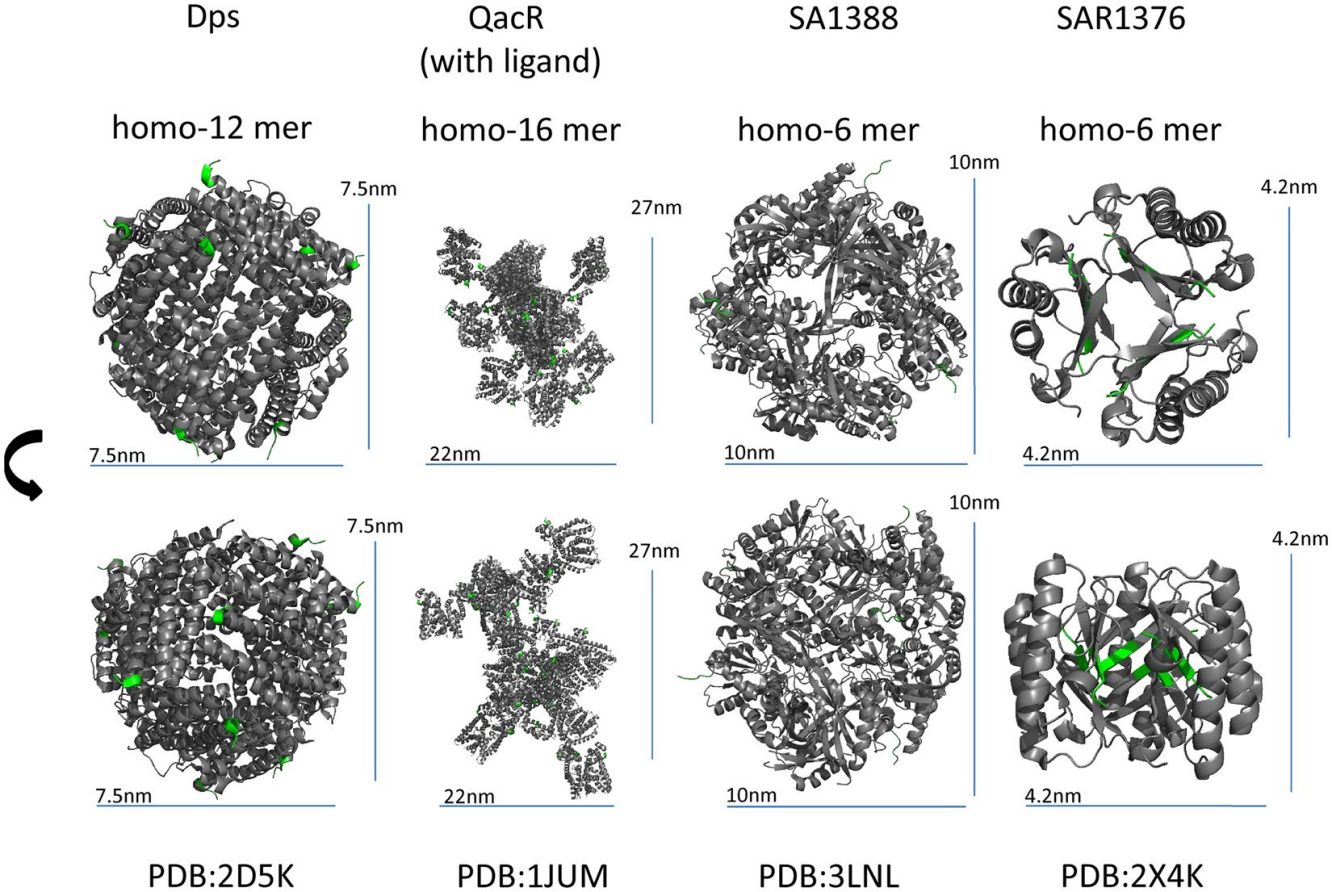
Crystal structures of four *S*. *aureus* multimerizing proteins, obtained from the Protein Data Bank. Gene identifiers are shown, together with two views of the molecule, rotated by 90°. The amino terminal of each protein, comprising five amino acids, is coloured green.

Study of crystal structures within the Protein Data Bank revealed a number of bacterial proteins which form self-multimers of various orders. A subset was selected based on absence of intra- or inter-chain disulphide bonding, absence of transmembrane regions and absence of toxicity and oncogenic activity, with a view to increasing the probability of efficient expression. In the first instance we chose to proceed with self-multimerising proteins from *S*. *aureus*, a pathogenic microbe causing disease that is controlled by both T cell and antibody mediated mechanisms^17^. Figure 1 shows the structures of the four protein chosen: Dps-like Peroxide Resistance Protein Dpr, with a structure similar to that of ferritin^12^, QacR, a multidrug binding transcriptional repressor, SA1388^18^, a protein of unknown function annotated as a homologue of *E*. *coli* ygbL aldolase class 2-like gene, and SAR1376, a 4-Oxalocrotonate Tautomerase (4-OT). The size of the crystallising unit varied from more than 20 nm (for QacR) to less than 5 nm (for SAR1376) (Fig. 1).

### Fusion of SAR1376 to *S*. *aureus* antigens enhances their immunogenicity in mice

**Figure 2:**
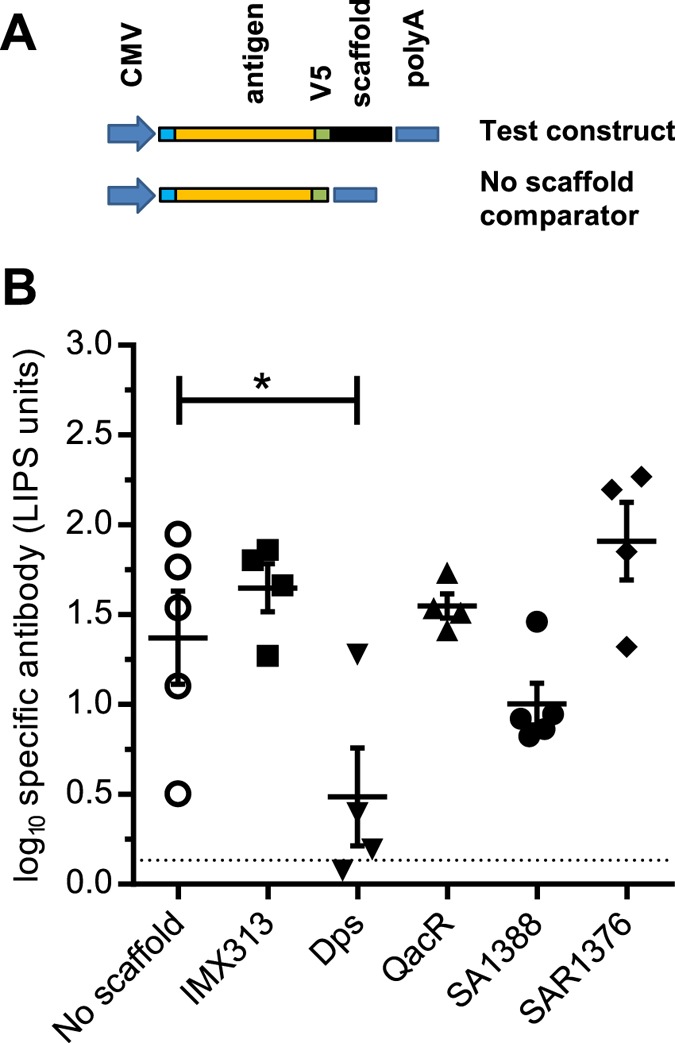
Design and immunogenicity of DNA vectors expressing the *S*. *aureus* BitC antigen fused to the novel multimerising scaffolds. (**A**) Design of pMono2 DNA vaccination vectors constructs. CMV: CMV IE94 promoter, TPA: human tissue plasminogen activator signal sequence, antigen: antigen being tested, V5: epitope tag and linker sequence, scaffold: multimerising domain, BGH pA: Bovine growth hormone polyadenylation sequence. (**B**) BitC-specific IgG response after two immunisations of groups of 4 or 5 BALB/c mice with 50 µg BitC-scaffold DNA two weeks apart, measured by LIPS assay at 3 weeks post boost. Each symbol represents a single mouse. Dotted line: threshold for background response, *p < 0.05.

We hypothesised that these four bacterial molecules might have pro-immunogenic activity similar to other multimerising scaffold proteins, and so might enhance immune responses to antigens fused to them^2, 12, 13^. Expression vectors producing fusions of the four proteins (Table 1) with a series of *S*. *aureus* antigens were constructed. The expression cassette was composed of a human tissue plasminogen activator leader sequence, the antigen of interest, an epitope tag (V5) used to monitor protein expression, and the scaffolding domain (Table 1) via a GSG linker (Fig. 2A).

**Table 1:** DNA sequences of multimerising proteins investigated. All are human codon optimized.

Dps	ATGAGCAACCAGCAGGACGTCGTGAAAGAACTGAATCAGCAGGTGGCCAACTGGACCGTGGCCTACACCAAGCTGCACAACTTCCATTGGTACGTGAAGGGCCCCAACTTCTTCAGCCTGCACGTGAAGTTCGAGGAACTGTACAACGAGGCCAGCCAGTACGTGGACGAGCTGGCCGAGAGAATCCTGGCCGTGGGCGGAAATCCTGTGGGCACCCTGACCGAGTGCCTGGAACAGAGCATTGTGAAAGAGGCCGCCAAGGGCTACAGCGCCGAGCAGATGGTGGAAGAACTGAGCCAGGACTTCACCAACATCAGCAAGCAGCTGGAAAACGCCATCGAGATCGCCGGCAACGCTGGCGACGATGTGTCCGAGGACATGTTCATCGGCATGCAGACCAGCGTGGACAAGCACAACTGGATGTTCAAGAGCTACCTGAGCTGATGATGA
QacR	ATGAACCTGAAGGACAAGATCCTGGGCGTGGCCAAAGAGCTGTTCATCAAGAACGGCTACAACGCCACCACCACCGGCGAGATCGTGAAGCTGAGCGAGAGCAGCAAGGGCAACCTGTACTACCACTTCAAGACCAAAGAGAACCTGTTCCTGGAAATCCTGAACATCGAGGAATCCAAGTGGCAGGAACAGTGGAAGAAAGAACAGATCAAGTGCAAGACCAACCGCGAGAAGTTCTACCTGTACAACGAGCTGAGCCTGACCACCGAGTACTACTACCCCCTGCAGAACGCCATCATCGAGTTCTGCACAGAGTACTACAAGACCAATAGCATCAACGAGAAGATGAACAAGCTGGAAAACAAGTACATCGACGCCTACCACGTGATCTTCAAAGAGGGCAATCTGAACGGCGAGTGGTGCATCAATGACGTGAACGCCGTGTCCAAGATCGCCGCCAACGCCGTGAATGGCATCGTGACCTTCACCCACGAGCAGAACAT CAATGAGCGGATCAAGCTGATGAACAAATTCAGCCAGATCTTCCTGAACGGCCTGAGCAAGTGATGA
SAR1376	ATGCCCATCGTGAACGTGAAGCTGCTGGAAGGCAGAAGCGACGAGCAGCTGAAGAACCTGGTGTCCGAAGTGACCGACGCCGTGGAAAAGACCACCGGCGCCAACAGACAGGCCATCCACGTCGTGATCGAGGAAATGAAGCCCAACCACTACGGCGTGGCCGGCGTGCGGAAAAGCGATCAGTGATGA
SA1388	ATGAAGATCGCCGACCTGATGACCCTGCTGGACCACCACGTGCCCTTTAGCACAGCCGAGAGCTGGGACAACGTGGGCCTGCTGATTGGCGACGGCGACGTGGAAGTGACCGGCGTGCTGACAGCCCTGGACTGCACACTGGAAGTCGTGAACGAGGCCATCGAGAAGGGCTACAACACCATCATCAGCCACCACCCCCTGATCTTCAAGGGCGTGACCAGCCTGAAGGCCAACGGCTACGGCCTGATCATCCGGAAGCTGATCCAGCACGACATCAACCTGATCGCCATGCACACCAACCTGGACGTGAACCCCTACGGCGTGAACATGATGCTGGCCAAGGCCATGGGCCTGAAGAACATCAGCATCATCAACAACCAGCAGGACGTGTACTACAAGGTGCAGACCTACATCCCCAAGGATAATGTGGGCCCCTTCAAGGACAAGCTGAGCGAGAATGGCCTGGCCCAGGAAGGCAACTACGAGTACTGCTTCTTCGAGAGCGAGGGCAGAGGCCAGTTCAAGCCTGTGGGCGAGGCCAACCCTACCATCGGCCAGATCGACAAGATCGAGGACGTGGACGAAGTGAAGATCGAGTTCATGATCGACGCCTACCAGAAGTCCAGAGCCGAGCAGCTGATCAAGCAGTACCACCCCTACGAGACACCCGTGTTCGACTTCATCGAGATTAAGCAGACCTCCCTGTACGGCCTGGGCGTGATGGCCGAGGTGGACAACCAGATGACTCTGGAAGATTTCGCCGCCGACATCAAGAGCAAGCTGAACATCCCCTCCGTCAGATTCGTGGGCGAGAGCAACCAGAAGATCAAGCGGATCGCCATCATCGGCGGCAGCGGCATCGGCTACGAGTATCAGGCTGTGCAGCAGGGCGCCGACGTGTTCGTGACAGGCGATATCAAGCACCACGACGCCCTGGACGCCAAGATCCATGGCGTGAACCTGATCGACATCAACCACTACAGCGAGTACGTGATGAAGGAAGGCCTGAAAACCCTGCTGATGAACTGGTTCAATATCGAGAAGATTAACATTGATGTGGAAGCCAGCACCATCAATACCGACCCCTTCCAGTACATCTGATGATGA
SAR1376 P1A*	ATG**GCC**ATCGTGAACGTGAAGCTGCTGGAAGGCAGAAGCGACGAGCAGCTGAAGAACCTGGTGTCCGAAGTGACCGACGCCGTGGAAAAGACCACCGGCGCCAACAGACAGGCCATCCACGTCGTGATCGAGGAAATGAAGCCCAACCACTACGGCGTGGCCGGCGTGCGGAAAAGCGATCAGTGATGA
SAR1376 R35A*	ATGCCCATCGTGAACGTGAAGCTGCTGGAAGGCAGAAGCGACGAGCAGCTGAAGAACCTGGTGTCCGAAGTGACCGACGCCGTGGAAAAGACCACCGGCGCCAAC**GCC**CAGGCCATCCACGTCGTGATCGAGGAAATGAAGCCCAACCACTACGGCGTGGCCGGCGTGCGGAAAAGCGATCAGTGATGA

*Mutation in bold, underlined.

We studied scaffold fusions to two *S*. *aureus* antigens: BitC^19^, a cell surface lipoprotein (accession NP_370379) which we have previously investigated as a vaccine candidate (unpublished data, patent 14/433565), and the extracellular domain of the Clumping factor B precursor^20^ (ClfB, accession YP_001333563). As comparators, constructs expressing antigen without scaffold were made (Fig. 2A). Additionally, in some experiments, the multimerising tag IMX313^2^ fused to the antigen C-terminus was used as a positive control.

Groups of BALB/c mice were immunised intramuscularly with a mammalian expression DNA vector expressing BitC fused to one of the four test scaffolding domains. A priming and boosting immunisation was administered, separated by two weeks. Measurement of the antibody response against BitC three weeks post-boost indicated that fusion of SAR1376 might increase immunogenicity of BitC relative to the no-scaffold comparator (*p* = 0.16), while the mice vaccinated with the Dps scaffold responded poorly against BitC (Fig. 2B). In this experiment, the fold change in antibody concentrations (if any) was small.

**Figure 3:**
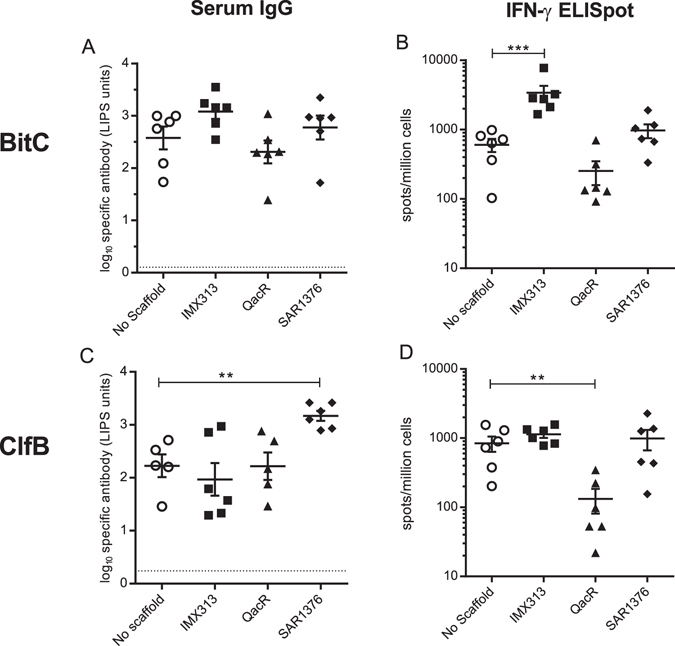
SAR1376 scaffold enhances immune responses to BitC and ClfB in BALB/c mice. Immunogenicity was assessed following prime-boost vaccination with DNA constructs encoding different scaffolds (as indicated on the X axes) fused to the antigen C-terminus; antigen specific IgG responses against BitC (panel A) and ClfB (panel C) measured by LIPS assay, and number of IFN-γ producing T-cells induced by BitC (panel B) and ClfB (panel D) at week 3 post boost. Each symbol represents a single mouse. Dotted line: threshold for background response. **p < 0.01, ***p < 0.001.

We further tested the SAR1376 and QacR scaffolds with both ClfB *S*. *aureus* antigen and with BitC, in the same 2-week prime-boost vaccination regimen. Humoral and cellular (IFN-γ ELISpot) responses were measured following the second immunisation. Comparator constructs containing the QacR domain were included in order to assess the specificity of the observed response. The immunogenicity results supported the previous experiment, in which a small increase was observed in antibody responses BitC when BitC was linked to SAR1376 but not QacR (Fig. 3A; *p* = *0*.*06* for BitC-SAR1376 when pooling data from experiments shown in Figs 2 and 3). A significantly higher antibody response was observed to ClfB was observed using ClfB-SAR1376 compared to ClfB without a scaffold (*p* = 0.002, Fig. 3C). This experiment allowed comparison of the fold change elicited in antibodies against two antigens, BitC (where significant changes were not observed) and ClfB, where an estimated 10 fold change (2.2 log_10_ units vs. 3.2 log_10_ units, p = 0.002) was observed. The SAR1376 scaffold did not enhance IFN-γ producing T-cell numbers with either of these two antigens (Fig. 3B and D), although the IMX313 tag, used here as a positive control, did increase the T cell responses to BitC (Fig. 3B, *p* = *0*.*01*).

### The 4-OT family

**Figure 4:**
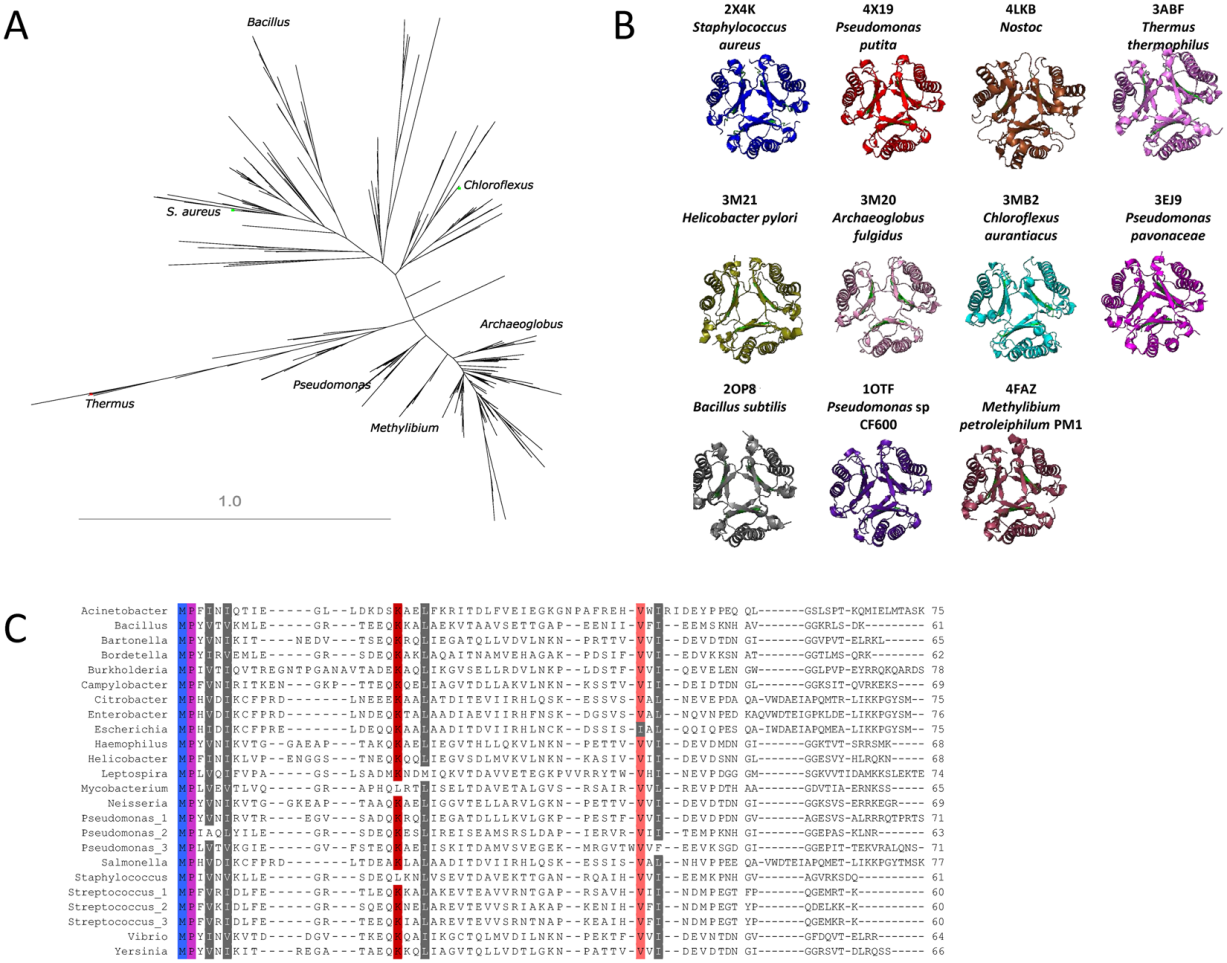
4-OT family members. (**A**) Family tree, 2780 discrete family members (modal length of 63 amino acids) were found across *Bacteria*. (**B**) Despite the extensive primary amino acid sequence diversity observed, crystal structures are very similar. (**C**) Cobalt alignment of 4-OT like sequences from selected genera which include human pathogens.

4-OT-like enzymes are common in bacteria^21^. Using a protein-based search strategy, 2780 discrete family members (modal length of 63 amino acids) were found across *Eubacteria*, with examples in *Archaea* also noted (see Supplementary Data S1). One example was chosen randomly from the 342 different genera identified (Supplementary Data S1). Extensive diversity is observed within the protein family, with only 20% identity in primary protein sequences between diverse members of the family (Fig. 4A). Examination of eleven bacterial crystal structures, however, show a very similar crystal structures in family members despite huge evolutionary distance (Fig. 4B, and also Supplementary Movie 2). Conserved motifs, including a highly conserved initial proline, do exist within the primary sequences from genera known to be pathogenic to man (Fig. 4C, see also Supplementary Data S1).

The crystal structure of SAR1376 reveals a hexamer forming an approximately spherical structure of about 5 nm diameter. It further suggests that the amino terminus of a short linker attached to the N-terminus of SAR1376 is surface accessible. Three such amino termini are present on each side of the sphere (Supplementary Movie S1). This suggests a model in which fusion of antigens to the N-terminus of SAR1376 generates a small sphere with six antigens displayed outwards (Fig. 1, Supplementary Movie S1).

### Inactivated SAR1376 enzyme retains adjuvant activity

**Figure 5:**
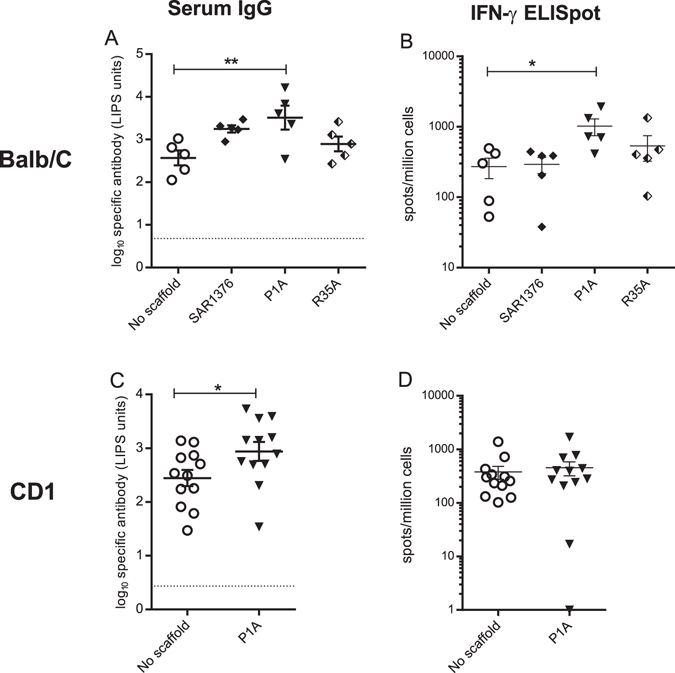
SAR1376 mutant P1A scaffold enhances immune responses to ClfB in BALB/c and CD1 mice. Mice were vaccinated twice with DNA vectors expressing the antigens shown. Antigen specific antibody responses against ClfB in BALB/c (panel A) and CD1 (panel C) mice as measured by LIPS assay, and number of IFN-γ producing T-cells induced by ClfB in BALB/c (panel B) and CD1 (panel D) mice after vaccination with mutant scaffolds fused to the c-terminus of the antigen. Sera were collected 3 weeks post second injection. Each symbol represents a single mouse. Dotted line: threshold for background response. *p < 0.05; **p < 0.01.

Since the mechanism of 4-OT catalysis has been heavily investigated, we mutated two critical residues involved in the active site, Proline-1 (P1) and arginine-35 (R35), a site corresponding to R39 in other crystallised family members^21^. P1A mutations disrupt enzymatic activity, but leave the protein structure intact, whereas R35A or Q mutations disrupt catalysis and impair protein multimerisation^21^. The immunogenicity of ClfB fused to these variants was compared (Fig. 5). The SAR1376 mutant P1A enhanced both the antibody and T-cell responses to ClfB significantly compared to no scaffold (*p* = 0.021) (Fig. 5A and B). By contrast, the immune response to ClfB with the scaffold containing the R35A mutation was not significantly different from ClfB without a scaffold (Fig. 5A and B). The effect of SAR1376 mutant P1A on the ClfB antibody response was similar in both BALB/c (Fig. 5A) and outbred CD1 mice (Fig. 5C). Enhancement on IFN-γ producing T-cell numbers by P1A was observed in one mouse strain (Fig. 5B and D). Taken together, this suggests that protein multimerisation contributes to the enhanced immunogenicity, but catalytic activity does not.

### SAR1376 and the P1A variant are immunogenic

**Figure 6:**
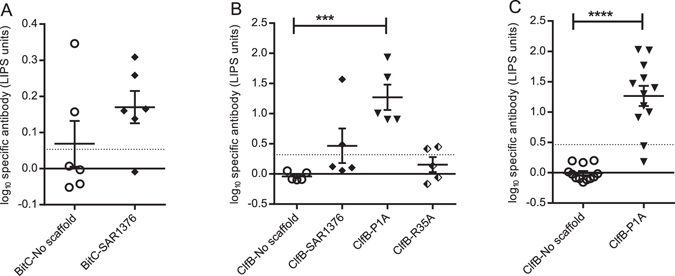
SAR1376 specific antibodies measured after vaccination with DNA vectors encoding the ClfB antigen with our without scaffold fused to the C-terminus of the antigen. (**A**) Antibody response against SAR1376 in BALB/c mice immunised with BitC-SAR1376 fusion protein. (**B**) Anti-SAR1376 antibody levels after vaccination of BALB/c mice with ClfB fused to SAR1376, SAR1376-P1A or SAR1376-R35A. (**C**) Anti-SAR1376 antibodies in CD1 mice immunised with ClfB-SAR1376 mutant P1A scaffold, as measured by LIPS assay. Each symbol represents a single mouse. Dotted line: three s.d. above assay background. ***p < 0.001; ****p < 0.0001.

We next investigated the capacity of SAR1376 fusion proteins to raise a humoral response against SAR1376 itself. Antibody responses against SAR1376 fused to either BitC (Fig. 6A) or ClfB (Fig. 6B) in immunised BALB/c mice were not significantly raised, as compared to the no scaffold groups. By contrast, immunization with ClfB fusion constructs containing the SAR1376 mutant P1A scaffold raised a significant antibody response to SAR1376 in both BALB/c (Fig. 6B) and CD1 outbred (Fig. 6C) mice whereas the R35A mutation did not (Fig. 6B). In both strains, serological responses against SAR1376 in the absence of SAR1376 immunisation were indistinguishable from the assay background (Fig. 6), suggesting that SAR1376 homologues, if present in colonising murine microbiota, do not generate detectable cross reactivity with SAR1376.

### Viral Vectored vaccines expressing SAR1376-P1A fused to truncated Hla increase immunogenicity

**Figure 7:**
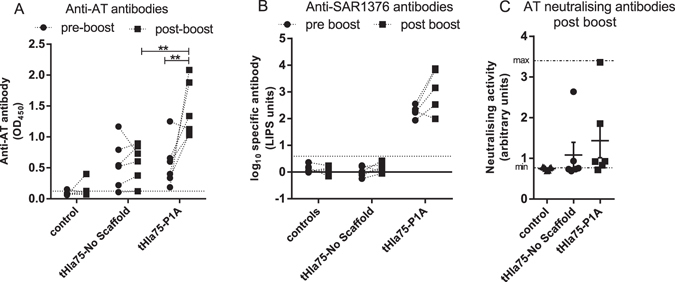
Impact of fusion of SAR1376 mutant P1A to truncated Hla on immunogenicity. (**A**) Anti-AT antibody levels. (**B**) Anti-SAR1376 antibody levels after vaccination of BALB/c mice with tHla75-no scaffold, tHla75-P1A, or empty vectors (control) as assessed respectively by ELISA and LIPS assay. Sera were collected pre boost (day 56 post Adenovirus prime) and post MVA boost (day 70 post prime). Dotted line: threshold for background response. (**C**) AT neutralizing activity of the antibodies was assessed post boost by Toxin Neutralisation Assay. Assay minimum and maximum levels were determined with mAb 8B7 and recombinant AT as described in Methods. **p < 0.01.

We investigated whether the pro-immunogenic effect of SAR1376 fusion was restricted to DNA vaccination. Because the effect of SAR1376 fusion appeared most marked on antibody induction, we elected to study the *S*. *aureus* alpha toxin (AT), a haemolytic multimeric β-pore forming toxin which is a critical virulence factor in *S*. *aureus* ^22^ and is encoded by the *Hla* gene. AT can be neutralised by antibody^22^. We designed a truncated form of AT, designated tHla75, comprising amino acids 1–75, the portion of the molecule reported to contain the receptor ADAM10 (A disintegrin and metalloproteinase 10) binding domain^23, 24^. Recombinant adenoviral (AdH5) and MVA vectors expressing SAR1376 fused to tHla75 were constructed. BALB/c mice were vaccinated with AdH5-tHla75 followed eight weeks later by MVA-tHla, a prime-boost regime known to be highly immunogenic^2^. Analysis of the immune response against alpha toxin showed that SAR1376 fusion did not increase the immunogenicity of adenovirally expressed proteins (Fig. 7A), but two weeks post MVA boost a significantly enhanced antibody response was observed in the tHla75-P1A group relative to the tHla75-no scaffold group (Fig. 7A). However, the tHla75 construct did not induce substantial functional (neutralising) antibody titres (Fig. 7C) in either configuration. As expected, antibodies against SAR3176 were raised and boosted in tHla75-P1A vaccinated group animals (Fig. 7B).

In summary, fusion of *S*. *aureus* antigens to SAR1376-P1A significantly increased antibody responses to the antigens, when expressed from DNA vaccine vectors and viral vectors. The enhancement was observed in two different mouse strains, and was abrogated by a mutation known to disrupt multimerisation of SAR1376. Baseline immune responses against SAR1376 were not detected in the two mouse strains, but SAR1376-P1A variant is itself immunogenic.

### Fusion of SAR1376-P1A to Pfs25 recombinant protein improves immunogenicity in BALB/c mice

We investigated whether the pro-immunogenic effect of SAR1376 fusion extended to recombinant protein antigens by studying the effect SAR1376 fusion on immunogenicity of a *P*. *falciparum* protein, Pfs25. Pfs25 is a candidate antigen for a transmission blocking vaccine and antibodies against Pfs25 have been shown in several studies to interfere with sexual reproduction of the parasite in the mosquito vector^25^. Recently, we have reported that heptamerisation of Pfs25 by fusion to IMX313 increased its immunogenicity significantly in pre-clinical studies^10^.

**Figure 8:**
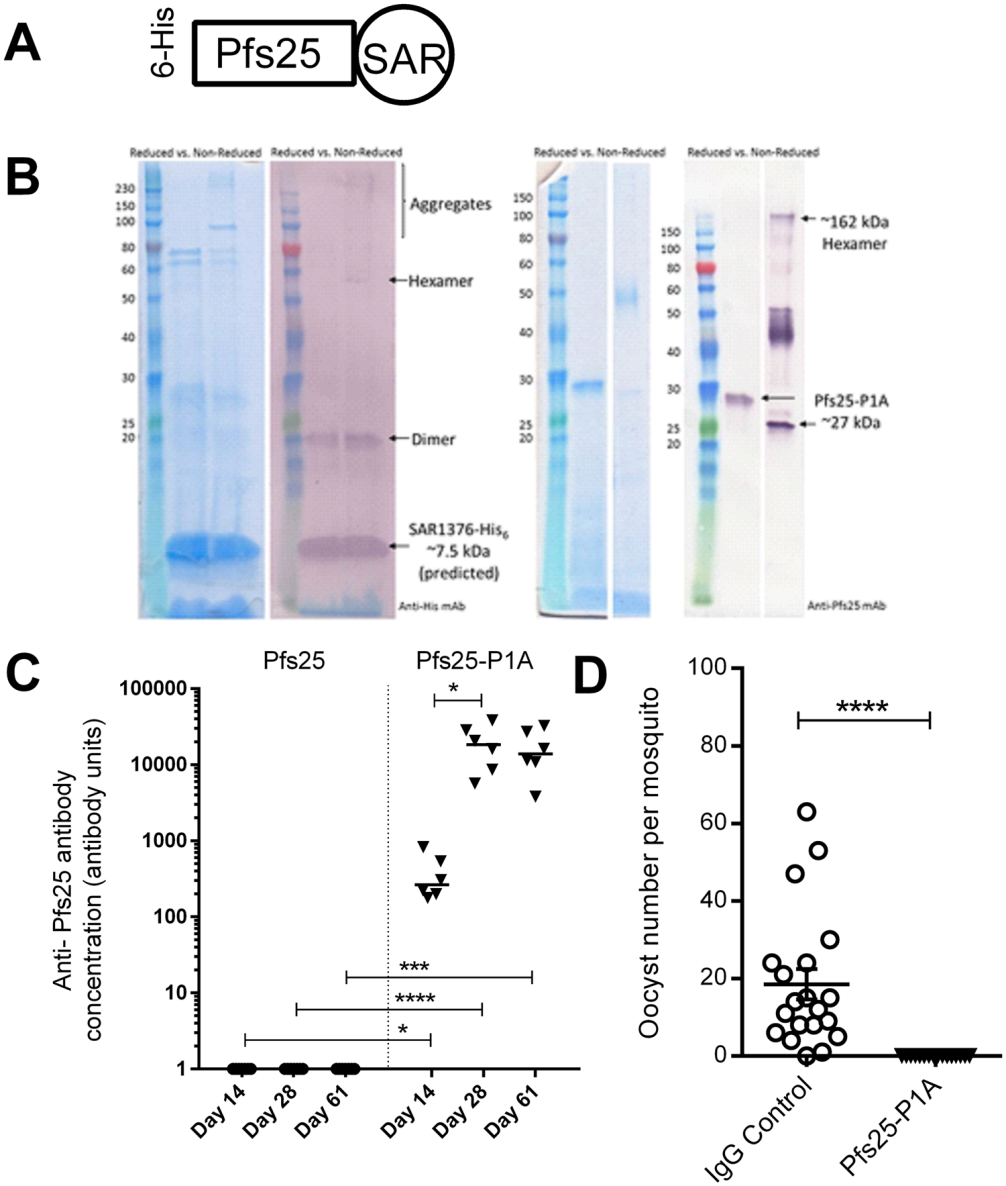
Fusion of SAR1376-P1A to Pfs25 improves functional activity. (**A**) Plasmid construct for expression of pfs25-SAR1376 P1A in *Pichia pastoris*. (**B**) SDS page and Western Blot of SAR1376 (anti-His mAb), and Pfs25-SAR1376-P1A (anti Pfs25 mAb). (**C**) Antibody response after vaccination of BALB/c mice with monomeric Pfs25 or Pfs25 fused to SAR1376-P1A. Sera were collected at day 14, 28 and day 61 post prime and IgG responses measured using a standardised ELISA. Each solid symbol represents a single mouse. (**D**) Pooled-purified IgG mixed with *P*. *falciparum* gametocytes was fed to *Anopheles stephensi* mosquitoes through a membrane. Midguts were dissected 9 days post-feed and the number of oocysts per gut were counted. Each symbol represents the count from one mosquito. AU = antibody units. *p < 0.05; ***p < 0.001; ****p < 0.0001.

Recombinant monomeric Pfs25 and Pfs25-SAR1376-P1A proteins were produced in *Pichia pastoris* as secreted proteins and purified using a 6-Histidine tag (Fig. 8A and B). Mice were immunised twice with either Pfs25-SAR1376-P1A or monomeric Pfs25 protein-in-Alhydrogel formulations at 2-week intervals.

Little response was seen in mice following vaccination with Pfs25. This is not unexpected as we have previously observed that monomeric protein is poorly immunogenic using Alhydrogel adjuvant^10^. The antibody levels at all time points were significantly higher in the group that received Pfs25-SAR1376-P1A than the mice receiving monomeric Pfs25, demonstrating that fusion of Pfs25 to SAR1376-P1A significantly improved the immune response (Fig. 8C). The pooled and purified IgG from the Pfs25-SAR1376-P1A immunised mice (day 28) were able to completely block oocyst development in the mosquito midgut in the *ex vivo* Standard Membrane Feeding Assay (SMFA), demonstrating that antibodies produced by immunisation with this platform were functional (Fig. 8D).

## Discussion

In this study we show that fusion of the 4-OT enzyme family member SAR1376 to different antigens enhances antibody responses against the fused antigen. The effect was demonstrated using three vaccine delivery platforms: DNA, viral vector delivery systems and recombinant proteins, and several antigens: three from *S*. *aureus* and one from *Plasmodium falciparum*. For some proteins, the fold change in antibody levels observed was modest (such as the lipoprotein BitC, where less than 2-fold change was observed), but for others (such as Pfs25, and ClfB) more substantial enhancements were observed. In the case of *P*. *falciparum* Pfs25, we could demonstrate that this increase was associated with a functional antibody activity.

4-Oxalocrotonate Tautomerase (4-OTs), of which *S*. *aureus* enzyme SAR1376 is an example, are typically 60–80 amino acids in length, placing them among the smallest enzymes known. They have an unusual mechanism of action, involving the proline at residue 1 (after the initiator methionine) and are involved in the catalytic breakdown of polycyclic compounds into tri-carboxylic acid (Krebs’) cycle precursors in a variety of bacteria^21^. A range of other enzymatic activities have been described in proteins with 4-OT-like structures^21, 26^, but all depend on the initial proline. By contrast, the enhancement of immunogenicity observed here does not depend on the initial proline, and is in fact enhanced following P1A mutation, suggesting that enzymatic activity is not required for the adjuvant effect.

The ability of 4-OT proteins to spontaneously multimerise into hexamers was exploited to enhance the immune response to several different antigens. This ability is not compromised by the ligation of the antigen of interest via a short linker to the N-terminus of the enzyme, as judged by crystal structures in which the N-terminus of the linker remains surface-exposed in the resulting hexamer (Movie S1). The results of our mutagenesis experiments support the idea that multimerisation of SAR1376 is required for enhanced immunogenicity. The mechanism by which this multimerisation increases immunogenicity was not investigated but is most likely due to the particulate nature of the resulting protein and/or pattern recognition of the repetitive antigen arrangement on the hexamer surface, as described with other multimerising ligands^4^. It is possible that such multimerisation has a larger impact on the immunogenicity (such as Pfs25) than on others (such as BitC), perhaps because immune detection of the native proteins varies, with the impact of additional multimerisation more evident in poorly immunogenic proteins.

A range of other *S*. *aureus* proteins reported to multimerise were also tested for pro-immunogenic activity (Dps, QacR, SA1388) using the DNA vaccination system which detected the activity of SAR1376. None displayed a similar effect. This is surprising given the structural similarities between Dps and ferritin, a self-multimerising molecule which is successful at increasing immunogenicity to some antigens when fused to their C-terminus^12^. It is possible that of the proteins examined, only SAR1376 adopts a pro-immunogenic, likely multimeric, structure *in vivo* when produced by the expression systems we studied.

Of note, 4-OTs are widespread in pathogenic bacteria^27^. There are thousands of sequences known from multiple bacterial species, both Gram positive and Gram negative. Given their conserved structure, inactivating the enzymatic activity while retaining ability to multimerise and fusion of diverse bacterial 4-OT proteins may be a general strategy for enhancing antibody responses to vaccine antigens. In the development of novel vaccines the approach combining potent viral vectors with a new generation of short, multimerising approach could represent a promising alternative to virus like particles, which have recently become an attractive vaccination platform^4^, as it combines the inherent immunogenicity and safety of viral vectored vaccines^28^ with stable spherical structures surface-displaying the antigen.

## Methods

### Scaffolds used and their production

Chosen scaffold sequences where human codon optimised and DNA synthetized (GeneArt, Life Technologies Ltd) (Table 1). Scaffolds were fused to the C-terminus of the *S*. *aureus* antigens in a mammalian expression vector (pMono2) using a restriction enzyme based strategy. Antigens fused were *S*. *aureus* BitC, a *S*. *aureus* cell surface lipoprotein (accession NP_370379) which we have previously investigated as a vaccine candidate (unpublished data, patent 14/433565), the extracellular domain of the *S*. *aureus* Clumping factor B precursor^20^ (ClfB, accession YP_001333563), *S*. *aureus* α–hemolysin (accession YP_111574996, amino acids 1–75, designed tHla75 here), and *P*. *falciparum* protein Pfs25 (accession AAN35500)^10^. Sequences for all these were synthesised by Life Technologies Ltd.

*S*. *aureus* tHla75 was ligated into the pMono2 vector from which the construct was subcloned into shuttle vectors and transfected into replication-deficient adenovirus human serotype 5 (AdHu5) and Modified Vaccinia Ankara (MVA) as described elsewhere^29, 30^.

The gene coding for *Plasmodium falciparum* transmission-blocking antigen, Pfs25 with a 6-his tag (His_6_-Pfs25) was fused to the 5′ upstream of the gene coding for SAR1376-P1A and ligated into the pPinkα-HC plasmid (Fig. 8A) which puts protein expression under methanol inducible control and allows secretion of the expression product from *Pichia pastoris* via the α-mating factor secretion signal. Electrocompetent *P*. *pastoris* were transformed with the expression plasmid. Colonies were screened for optimal expression and the highest expressing clone selected for scale up. A one litre shake flask culture of the selected clone was grown under inducing conditions and the supernatant harvested. Supernatant containing the secreted expression product was harvested by centrifugation and the product purified by nickel-chelate affinity chromatography.

### Vaccination experiments

All mouse procedures were conducted in accordance to the Animal (Scientific Procedures) Act 1986 (Project licence 30/2825) and were approved by the University of Oxford Animal Care and Ethical Review Committee. Six to eight week old female BALB/c or CD1 mice from Harlan Laboratories UK were used.

***In DNA vaccination experiments***, groups of 4-12 mice (BALB/c or CD1) were immunised intramuscularly with 50 μg vector DNA in 50 μl PBS (25 µl/hind leg). Immunisation was repeated 2 weeks later. On day 35, blood samples were taken from all animals under terminal anaesthesia (heart bleeds) for immune assays (IFN-gamma secreting T-cell numbers (ELISpot), and antibody levels by Luciferase ImmunoPrecipitation System (LIPS) assay)^31, 32^.

***For viral vector immunization***, groups of 6 mice were immunised intramuscularly with 10^9^ i.u. AdHu5 in 25 μl PBS followed at least 8 weeks later by 10^7^ pfu MVA as prime-boost sequence, a regime we refer to as AM7. Venous blood samples were taken from the tail vein of all animals pre boost and 2 weeks post boost.

***For immunization with recombinant proteins***, groups of 6 BALB/c mice were immunised intramuscularly with 50 µl aliquots (25 µl/hind leg) of protein-in-Alhydrogel formulations, containing 2.5 µg of either Pfs25-P1A or monomeric Pfs25 twice at 2 week intervals. Blood was collected from the tail vein on day 14 (2 weeks post prime) and day 28 (2 weeks post boost).

### Assessment of immune responses against BitC and ClfB

A Luciferase ImmunoPrecipitation System (LIPS) assay was used to detect specific serum anti-*S*. *aureus* BitC and ClfB antibodies as described^32^. Briefly, recombinant BitC and ClfB fusion protein with Renilla luciferase were produced in 293 cells as described^32^. Serially diluted sera were incubated with *Renilla* luciferase-BitC or ClfB fusion proteins. The mix was added to filter plates loaded with A/G beads (Thermo Fisher). After incubation and subsequent washings, chemiluminescence was measured in a Luminometer (ClarioStar, BMG Labtech) after adding substrate (Renilla luciferase assay system, Promega UK Ltd.). Log transformation was applied to luminescence data prior to statistical analysis. Specific luminescence was generated by subtracting the assay background, which was considered to be the luminescence observed in the absence of any sera. The assay limit of detection was considered to be four standard deviations above the specific luminescence in the control groups.

### Anti-alpha toxin immune responses

The anti-tHla antibody levels and functional activity (neutralizing activity, NA) of the antibodies in serum were assessed respectively by ELISA and Toxin Neutralisation Assay (TNA) as described by Oscherwitz and Cease^33^. In brief, the ability of antibody to block recombinant alpha toxin (AT) cytotoxicity *in vitro* was assessed using the Jurkat T cell line (TIB-152, ATCC, Manassas, VA). Mouse anti-Staphylococcal alpha hemolysin mAb (8B7) (IBT Bioservices # 0210-001) was used as standard positive to obtain minimum and maximum levels for neutralisation of AT (H9395, Sigma Biologicals).

### Anti-Pfs25 immune responses

Antibody levels in serum were assessed by standardised anti-Pfs25 ELISA, as described^10^. A serially diluted standard reference serum with a known antibody titre was used to determine the antibody titre of individual samples. Total IgG was purified from the pooled serum of the mice immunised with Pfs25-SAR1376-P1A and assessed by functional assay by Standard Membrane Feeding Assay (SMFA). This assay involves feeding malaria infected blood mixed with purified IgG to *Anopheles stephensi* mosquitos through a membrane^34^. If the IgG has functional activity it will block development of the malaria sexual stage in the mosquito midgut and at 9 days post feed there will be a reduction in the number of oocysts observed in the gut compared to a non-functional IgG control.

### Statistical analysis

Data on antibody response and IFNγ-specific spots were statistically analysed for effect of added scaffold by means of an *F*-test after a log_10_ transformation and correction for background. Log (number of IFN-γ secreting cells) was used, because of the approximate log-normal distribution of ELISpot counts in the animals (not shown). Specific antibody levels from the LIPS assay were generated by subtracting the assay luminescence background, which was considered to be the luminescence observed in the absence of any sera, from the luminescence observed with serum dilutions added. The assay limit of detection was considered to be four standard deviations above the background. Post-hoc pairwise comparisons were performed using Dunnett’s Multiple Comparison Test. Differences were considered significant when *p* < 0.05. The statistical packages used were R 2.15 (http://www.cran.org), and GraphPad Prism version 5.04 (GraphPad Software, Inc.).

### Bioinformatic identification of 4-OT-like proteins

The NCBI RefSeq database was queried using BLASTp and delta-BLAST^14^ using default parameters with the *S*. *aureus* 4-OT enzyme (YP_040781.1) as a query. Further searches were performed using distant hits and results pooled, and then filtered using custom R scripts to include hits encoding proteins of 55 to 85 amino acids. Manual curation was performed, and the sequence start of predicted proteins was trimmed to begin with MP, as a proline is present in position 2 of all canonical family members^21^, i.e. any amino acids purported to originate from upstream initiator codons were removed. A single sequence was selected per genus; using genus-specific sequences, an alignment was prepared using the NCBI Cobalt multiple alignment engine^35^ with default parameters. Additionally, a tree was constructed using PhyML^36^ using default parameters, and visualised using Archeopterix^37^ software.

### Presentation of crystal structures

Crystal structures of proteins of interest were downloaded from the Protein data bank. A single hexameric structure was isolated from each set of crystal data using Pymol v.1.8.2 for Windows. For comparison of multiple 4-OT crystals, structures were aligned using CEAlign (Pymol) using default parameters. Pymol was also used to render images.

## Electronic supplementary material

Crystal structure of SAR1376

Crystal structures of SAR1376 homologues

Supplementary Information
