# Supplementary material for: The S. aureus 4-oxalocrotonate tautomerase SAR1376 enhances immune responses when fused to several antigens
Source: Sci Rep. 2017 May 11;7:1745. doi: 10.1038/s41598-017-01421-z (PMC5431793; doi:10.1038/s41598-017-01421-z)

The *S. aureus* 4-oxalocrotonate tautomerase SAR1376 enhances immune responses when fused to diverse antigens

Pauline M. van Diemen[1]
Darren B. Leneghan[2]
Iona J. Taylor[2]
Kazutoyo Miura[3]
Carole A. Long[3]
Anita Milicic[2]
Sumi Biswas[2]
Christine S. Rollier[4]
David H. Wyllie[1*]

Supplementary Information

S1 Video 1. The crystal structure of SAR1376. The crystal structure of SAR1376 reveals a hexamer forming an approximately spherical structure of about 5nm diameter. A linker attached to the N-terminus of SAR1376 is surface accessible: the N terminus of a GSG linker attached to the N-terminal proline is shown in green. Three such amino termini are present on each side of the sphere suggesting a model in which fusion of antigens to the N-terminus of SAR1376 generates a small sphere with six antigens displayed outwards.

S2 Video 2. Crystal structures of 4-OT family members. Extensive diversity is observed within the protein family, with only 20% identity in primary protein sequences. Despite huge evolutionary distance, family members show a very similar crystal structures.

S3 Supplementary Data 1. The 4-OT family. 4-OT-like enzymes are common in bacteria, 2780 discrete family members were found across *Eubacteria*, with examples in *Archaea* also noted. One example was chosen randomly from the 342 different genera identified.

1. Crystal Structure Of Native 4-ot From Pseudomonas Putida Mt-2 At 1.94 Angstrom [Isomerase, EC: 5.3.2.6]
Taxonomy: Pseudomonas putida
Proteins: 6 Chemicals: 1 modified: 2015-03-27
MMDB ID: 127738 PDB ID: 4X19

2. Crystal Structure Of A Putative 4-oxalocrotonate Tautomerase From Nostoc Sp. Pcc 120 [Isomerase]
Taxonomy: Nostoc sp. PCC 7120
Proteins: 6 Chemicals: 9 modified: 2013-08-09
MMDB ID: 112141 PDB ID: 4LKB

3. Crystal Structure Of Tomn, A 4-Oxalocrotonate Tautomerase Homologue In Tomaymycin Biosynthetic Pathway [Isomerase]
Taxonomy: Streptomyces achromogenes
Proteins: 6 modified: 2011-09-18
MMDB ID: 92760 PDB ID: 3RY0

4. Crystal Structure Of A 4-Oxalocrotonate Tautomerase Homologue (Tthb242)[Isomerase, EC: 5.3.2.2]
Taxonomy: Thermus thermophilus HB8
Proteins: 6 Chemicals: 3 modified: 2011-05-27
MMDB ID: 87295 PDB ID: 3ABF

5. Crystal Structure Of Dmpi From Helicobacter Pylori Determined To 1.9 Angstroms Resolution[Isomerase, EC: 5.3.2.-]
Taxonomy: Helicobacter pylori 26695
Proteins: 6 modified: 2011-10-10
MMDB ID: 84556 PDB ID: 3M21

6. Crystal Structure Of Dmpi From Archaeoglobus Fulgidus Determined To 2.37 Angstroms Resolution [Isomerase, EC: 5.3.2.2]
Taxonomy: Archaeoglobus fulgidus DSM 4304
Proteins: 6 modified: 2011-05-27
MMDB ID: 84555 PDB ID: 3M20

7. Crystal Structure Of Sar1376, A Putative 4-Oxalocrotonate Tautomerase From The Methicillin-Resistant Staphylococcus Aureus (Mrsa)[Isomerase]
Taxonomy: Staphylococcus aureus
Proteins: 6 Chemicals: 16 modified: 2011-05-27
MMDB ID: 83424 PDB ID: 2X4K

8. Kinetic And Structural Characterization Of A Heterohexamer 4-Oxalocrotonate Tautomerase From Chloroflexus Aurantiacus J-10-Fl: Implications For Functional And Structural Diversity In The Tautomerase ...
Taxonomy: Chloroflexus aurantiacus J-10-fl
Proteins: 6 Chemicals: 9 modified: 2012-12-28
MMDB ID: 82494 PDB ID: 3MB2

9. Structural And Mechanistic Analysis Of Trans-3-Chloroacrylic Acid Dehalogenase Activity[Hydrolase]
Taxonomy: Pseudomonas pavonaceae
Proteins: 6 modified: 2012-10-19
MMDB ID: 68292 PDB ID: 3EJ9

10. Crystal Structure Of The 4-Oxalocrotonate Tautomerase Homologue Dmpi From Helicobacter Pylori[Isomerase, EC: 5.3.2.-]

Taxonomy: Helicobacter pylori J99

Proteins: 6 modified: 2013-01-13

MMDB ID: 62347 PDB ID: 2ORM

---

11. Crystal Structure Of Ywhb- Homologue Of 4-Oxalocrotonate Tautomerase[Isomerase, EC: 5.3.2.-]

Taxonomy: Bacillus subtilis

Proteins: 6 modified: 2012-10-09

MMDB ID: 62221 PDB ID: 2OP8

---

12. 4-Oxalocrotonate Tautomerase-Triclinic Crystal Form[Isomerase, EC: 5.3.2.-]

Taxonomy: Pseudomonas sp. CF600

Proteins: 6 modified: 2013-01-12

MMDB ID: 4263 PDB ID: 1OTF

---

13. Kinetic And Structural Characterization Of The 4-oxalocrotonate Tautomerase Isozymes From Methylibium Petroleiphilum [Isomerase, EC: 5.3.2.-]

Taxonomy: Methylibium petroleiphilum PM1

Proteins: 6 Chemicals: 2 modified: 2014-03-27

MMDB ID: 110662 PDB ID: 4FAZ

S4 Supplementary Figure 1. Full size images of Figure 8B) SDS page and Western Blot of Pfs25-SAR1376-P1A (anti Pfs25 mAb). Sar refers to Sar1376. Rho and Tab are different 4-OT family members assayed in parallel, showing that other family members also produce multimeric forms.

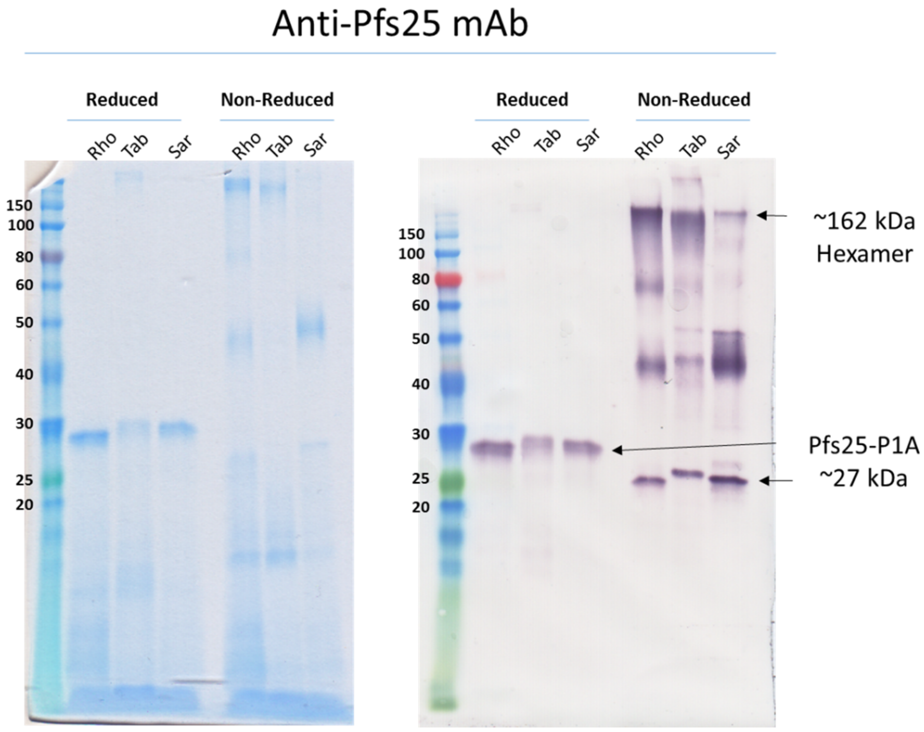

Supplement: Supplementary file 3 — Supplementary Information [file 41598_2017_1421_MOESM3_ESM.pdf]
